# The Resignation of Sir Edward Wood, J.P.

**Published:** 1914-12-05

**Authors:** 


### LEICESTER ROYAL INFIRMARY.

### The Resignation of Sir Edward Wood, J.P.

ON November 5, 1910, pages 171 to 174, we gave an exhaustive account of the eminent services rendered by Sir Edward Wood, the Chairman of this institution, and intimated that the scheme of reconstruction which he had carried out had brought his personality into prominence as a successful pleader for funds, the sum of £100,000 having been raised. Further, Sir Edward has built up the financial position of the Leicester Infirmary until it is to-day commensurate with a greatly enlarged expenditure due to the maintenance and continuous use of the additional buildings. To bring the institution quite up to a modern standard it was held essential to rebuild the Children's Hospital, the estimated cost of £8,000 being ultimately increased to £15,000. Sir Edward Wood had the satisfaction of carrying out this important work during his chairmanship, although, to his deep personal regret, ill-health made it impossible for him to be present at the opening ceremony in April last.

On his resignation Sir Edward Wood passes into well-earned retirement after decades of years' good work well done, but his example as an active and great hospital chairman should inspire the younger men of Leicester to go and do likewise. Sir Edward's letter of resignation is full of interest, and will, we hope, have a prominent place in the next annual report. We wish him many happy years full of pleasant memories and the society and inspiring companionship of a host of old friends and younger men who are learning to follow his example.

### THE BOARD OF GOVERNORS AND SIR EDWARD WOOD.

At a special meeting of the Board of Governors of the Leicester Infirmary on the 1st inst. Sir Edward Wood's resignation of the chairmanship was submitted.

Mr. C. J. Bond, the Deputy Chairman of the Board, in moving the acceptance of the resignation, paid a glowing tribute to the great abilities of Sir Edward Wood as a hospital chairman and administrator of great merit.

Mr. Fielding Johnson, jun. (the Chairman of the House Committee), seconded the resolution, which was supported in complimentary but regretful terms by many of the members of the board, and carried unanimously as follows:—

"That the Board of Governors of the Leicester Royal Infirmary has received Sir Edward Wood's intimation of his desire to resign the position of chairman of the board with the deepest regret. The board is deeply sensible of the greatness of the work which Sir Edward has accomplished at the Leicester Royal Infirmary during the last thirteen years. It desires to thank him most warmly for his unwearied efforts not only on behalf of the Infirmary, but also on behalf of the working men and women of Leicester and Leicestershire, so many of whom, as patients at the Infirmary, have benefited by his generosity, public spirit, and administrative ability. In expressing the earnest wish that Sir Edward's life may still be spared and his health restored, the members of the board are very gratified to think that they will still have the benefit of his wise advice and sympathetic interest in the welfare of the Infirmary."

### AN EXAMPLE OF CIVIC MERIT.

As chairman of the great local firm of Freeman, Hardy and Willis, Ltd., he built up a successful and thriving business at present employing hundreds of hands. As a member of the Town Council he rendered efficient public service and was four times elected mayor. He was for many years the vice-chairman of the Derwent Valley Water Board, and it was largely due to his foresight during his chairmanship of the Leicester Water Committee that Leicester became the most largely interested constituent municipality in this great scheme.

The Leicester Saturday Hospital Society, of which he is the president, owes its proud position largely to his sagacious guidance. From an income of £4,000 this fund has been so effectively organised that its finances have been gradually built up to last year's record total of nearly £16,000. Well, however, as he is known for his great public services, he is no less known as a munificent supporter of all charitable and philanthropic enterprise. There is scarcely a local charity list that does not include his name among its most liberal supporters. Indeed, many of his fellow-citizens will acknowledge that his sage advice and kindly counsel have been freely and disinterestedly given, to the lasting gain of those who have sought his opinion.

Finally, Sir Edward Wood's personal benefactions to the building fund of the Infirmary amount to many thousands of pounds, and when they are added to the amount subscribed by his firm the total makes a sum which nobly frames the inspiriting commendation of a friend and colleague, "He has by his generous gifts taught the people of Leicester how to give."

